# Induced vergence-accommodation conflict reduces cognitive performance in the Stroop test

**DOI:** 10.1038/s41598-018-37778-y

**Published:** 2019-02-04

**Authors:** François Daniel, Zoï Kapoula

**Affiliations:** IRIS Group, Physiopathologie de la Vision et Motricité Binoculaire CNRS FR3636, Université Paris Descartes, Paris, France

## Abstract

Interaction mechanisms between cognition and binocular motor control in reading saccades remain unclear. In this study we examine objectively saccades and fixations parameters during the Stroop test, involving three different levels of cognitive demand (reading, color denomination and interference). In addition, we experimentally induce accommodation and vergence conflicts during the different tasks. Twenty-one visually normal subjects (age 20.9 ± 1.45) performed the Stroop test in three different randomized conditions: a control normal viewing condition, a 16Δ base-out prism condition, and a −2.50D spherical lenses condition. Prisms and spherical lenses induced Vergence-Accommodation conflict. Eye movements were recorded with the Eyeseecam video-oculography device. The results show (1) longer fixation duration in the interference task than in the denomination task, and shorter fixation duration in the reading task; (2) a higher interference effect in the conflict induced conditions compared to the control condition; (3) a lower tolerance to prism induced conflict, with a higher destabilization of the binocular motor control of saccades and fixations. This suggests an interplay between vergence accommodation conflict and cognitive load: tolerance to the conflict seems to be lower in the more cognitively demanding interference Stroop task. The results consolidate the link between cognition and high quality of single binocular vision.

## Introduction

Cognitive executive functions, such as action planning, cognitive flexibility or decision making, represent high level processes responsible for the cognitive control of behavior and are known to be also related with academic achievement, especially working memory and inhibitory control that are essential for learning and maintaining attention^1–4^. Inhibition is an important dimension of cognition and refers to the capacity to inhibit dominant or automatic responses when necessary^1,5^. This executive function is closely linked to the capacity to control and focus attention. A golden test that enables to study cognitive executive functions such as inhibition and attention is the Stroop test^6,7^. Made up of different tasks such as reading and color denomination, the Stroop test highly involves the visual input to be completed, as the stimuli are exclusively visual. In this test, especially in the interference task, there is a succession of words designating different colors but printed in an incongruent color (for example the word “red” printed in green). The subjects must inhibit the automatic reading response in favor of a less obvious one: naming the color of the ink. The Stroop interference is used in neuroscience, in developmental studies including dyslexia, and in aging and neurodegenerative diseases to evaluate cognitive performances; particularly cognitive executive functions. It also shares common processes with reading (Protopapas *et al*.^8,9^).

In 2016^10^, we introduced a study of the link between cognitive processes and the accommodative and vergence evaluation. We demonstrated that asymptomatic subjects with no accommodative or binocular dysfunctions showed higher inhibition performances evaluated with the Stroop test compared to symptomatic subjects with convergence insufficiency. These results suggest that visual processes responsible for clear and single binocular vision could interfere with cognitive processes and attention deployment. However, the mechanisms involved remained unclear.

Recent neuroimaging studies identified a cortical network activated when inhibition is required that involves the prefrontal, parietal, temporal and cingulate areas^11,12^. These areas are similar to those implicated in the top-down attentional control, such as the anterior cingulate cortex (ACC), the intraparietal sulcus (IPS) and the dorsolateral prefrontal cortex^13–15^. The cerebellum appears to also be involved in attentional control and cognition^12,16,17^. Concerning the Stroop test, several neuroimaging studies showed an increase of activation in cortical areas such as DLPFC, ACC, posterior parietal cortex (PPC)^18–20^, that supports the previous statement concerning the link between the Stroop test, inhibition and attentional control. Note that the neural circuits that process vergence disparity, accommodation signals and saccadic eye movements implicate similar areas and networks (visual cortex, parietal and frontal lobes, cerebellum)^21–26^. Thus, it is plausible physiologically to expect some competition in sharing common resources by cognitive executive functions and process to obtain clear and single binocular vision.

From an experimental point of view, several optometric studies were designed to focus on the impact of an induced accommodation-vergence conflict on reading and cognitive performances. Narayanasamy *et al*.^27^. showed that a bilateral lens-induced hyperopia of 2.50D had a significant impact on academic-related performance in children. Indeed, subjects showed lower performances or longer time to accomplish the reading test. Moreover, this destabilization was exacerbated after 20 minutes of sustained near work, suggesting a negative impact of an induced higher accommodative demand on the cognitive performance. These results are in line with those of Garzia *et al*.^28^ in students: an accommodative stress-induced of −2.00D on each eye increased the time to accomplish a reading task. Similar results were also found in the study of Poltavski *et al*.^29^, showing that the same amount of stress induced on adults impacted not only the amount of the accommodative lag^30^, but also the performance in a neuropsychological task of sustained attention, as the reaction time was greater in the stress-induced condition compared to control. Ludlam and Ludlam^31^ used base-in prisms on students and demonstrated poorer performances in reading comprehension in the stress-induced condition compared to control. These results suggest that an accommodation/vergence conflict experimentally induced could be responsible on lower performance concerning cognitive processes linked to attention and inhibition. However, eye movements such as reading saccades and vergence were not investigated in these studies, and the neuropsychological tests used were different, depending on study design.

## Goals of the Study

The first goal of this study is to measure objectively eye movements with video-oculography during the different tasks of the Stroop test (reading, color denomination, interference). As the saccades are similar in the three tasks but the cognitive load differs, one could first expect changes of the pattern of exploration, changes in the binocular motor control such as saccade disconjugacy or in fixation stability (disconjugacy during the fixation).

The second goal of this study is to observe the potential impact of an induced vergence-accommodation conflict on the performances during the Stroop tasks. We expect that disturbing the usual balance between vergence and accommodation, using prisms or spherical lenses, will force the visual system to adapt its responses to keep a single and clear vision. This study will provide evidence to determine if such forcing will impact on the cognitive performance, on the binocular motor control of the saccades and fixations, or both interacting with each other. Moreover, comparing the incidence of a lens and a prism induced conflict will be finally possible, to establish whether blur or double vision is more affecting.

## Methods

### Subjects

A total of 24 voluntary students aged from 19 to 23 years (mean age 20.9 ± 1.45 years old, 11 males) who were studying optics at the Lycée d’Optique *Fresnel* in Paris, participated in this study. Three subjects reported constant diplopia when wearing the prisms and were therefore excluded of the sample. They followed an optometric screening using the same method as Daniel and Kapoula^10^. All selected subjects presented no binocular and/or accommodative dysfunctions using the norms established by Scheiman and Wick^32^ and by considering the number of signs used in the studies of Porcar and Martinez-Palomera^33^ and Shin *et al*.^34^. Subjects wore their habitual refractive correction (when necessary) to yield normal vision. Refractive errors (spherical equivalent) were ranged from −3.50D to +1.50D, and 11 subjects were wearing a correction before the experiment. The other inclusion criteria were: a minimum of 20/20 visual acuity for each eye, no signs of amblyopia or strabismus, stereoacuity under or equal to 40 arcsecs (evaluated with the Wirt Rings Stereo Test, Stereo Optical Company) and no neurological findings. In addition, subjects were excluded from the study if the following criteria were observed: vertical phoria >1 prism diopter (Δ); an antecedent of eye pathology or surgery that could affect visual acuity or motility; presence of a central suppression or a fixation disparity (checked with the Mallett Fixation Disparity Test Unit and the Mallett Near Vision Unit NV5); signs of color vision defects (checked with an Ishihara plate test), which would affect their ability to normally perform the Stroop test; constant double and/or blurry vision reported during the testing.

The investigation adhered to the tenets of the Declaration of Helsinki and was approved by the local human experimentation committee, the “Comité de Protection des Personnes” (CPP) Ile de France VI (No: 07035), Necker Hospital, in Paris. Written informed consent was obtained from all subjects after the nature of the procedure was explained.

### Procedure and testing

Every subject was sited in front of a computer screen, at 50 cm distance, and was asked to accomplish each task of the Stroop test in 3 different conditions. They were wearing a video-oculography EyeSeeCam system (University of Munich Hospital, Clinical Neuroscience, Munich, Germany, see http://eyeseecam.com/) and a trial frame (Oculus Adult UB3, Zeiss), with their usual correction if needed. Each task was preceded by a five-point calibration.

#### Stroop test

The version that we used was made up of 3 different tasks: in the “reading” task, the subject has to read aloud a succession of words designating colors (red, blue, green or yellow), written in black; in the “denomination” phase, the subject has to name a succession of dots of color (red, green, blue or yellow); in the “interference” phase, the subject has to name the color of the print of the word, printed in an incongruent color (red, green, blue or yellow), for example the word “red” printed in yellow. Each trial contains eighty items (10-line of 8-column matrix) spaced out 3.7 cm from each other (center to center, 4.2° at 50 cm distance) and placed randomly. Each letter of the words was about 0.4° of angular size. The diameter of the color dots was about 0.4° angular size as well. Subjects were instructed to finish as quickly as possible without making mistakes or omissions and tasks were randomized from one condition to the other. To minimize a potential training effect, a task could not be followed by the same one, even if the condition was also changing. Between two different trials, subjects were asked to close the eyes for one minute.

#### Conditions

Subject accomplished the Stroop test in 3 different conditions: (1) a Control condition, wearing their usual correction; (2) a Prism condition, wearing their usual correction and an 8Δ base-out prism placed on the trial frame and on each eye (about 2.5 Meter Angle, MA); (3) a Lens condition, wearing their usual correction and a −2.50D placed on the trial frame and on each eye. We chose these amounts of spherical or prism power to induce a similar amount of conflict on accommodative demand or convergence demand. To minimize potential training or fatigue effects, conditions were randomized differently for every subject.

### Assessment of visual and binocular functions

Optometric screening was done for all the subjects prior to the experiment and on separate day. We used similar methods and materials than Daniel and Kapoula^10^ to evaluate the visual functions in different areas: symptomatology (using the CISS^35^), visual acuity, binocular vision (stereo acuity, central suppression and fixation disparity), vergence (NPC, fusional ranges at far and near distance, vergence facility), accommodation (Binocular Fused Cross Cylinder, Negative and Positive Relative Accommodation, monocular and binocular Near Point of Accommodation, binocular and monocular accommodative facilities), phorias and AC/A ratio.

### Eye movements recording

The subject was asked to accomplish the different tasks, and eye movements were recorded binocularly with a video-oculography EyeSeeCam system (University of Munich Hospital, Clinical Neuroscience, Munich, Germany, see http://eyeseecam.com/). At the beginning of each task, a 5-points calibration sequence was run using a matrices of laser dots: a central dot and four peripheral dots displayed at 8.5° rightward, leftward, downward and upward. Subjects fixated each dot one by one for four times, and total calibration task lasted about 20 seconds.

### Eye movement analysis

Calibration factors for each eye were extracted from the saccades recorded in the calibration task. We used the network Analyze32 to extract and analyze the data. From the individual calibrated eye position signal, we derived the horizontal conjugate signal by calculating the mean of the two horizontal eye positions, i.e. (left eye + right eye)/2, and the horizontal disconjugate signal, by calculating the difference position between both eyes, i.e. left eye – right eye. The velocity of the horizontal conjugate and disconjugate signals were computed using a symmetrical two-point differentiator combined to low-pass filtering with a Gaussian FIR filter (cut-off frequency 33 Hz).

**Figure 1:**
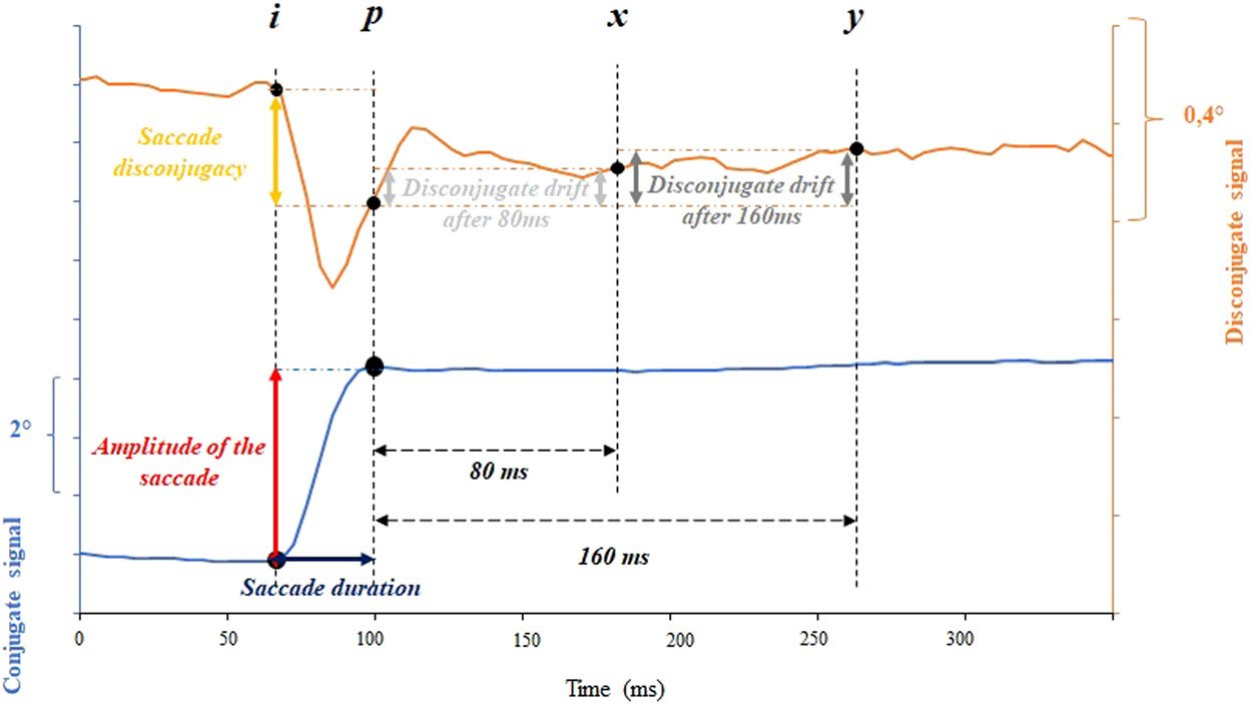
Analysis and marking of the reading saccades: determination of the saccade and of the fixation duration. ‘i’ and ‘p’ indicate respectively the beginning and the end of each saccade. We studied the post saccadic drift 80 ms and 160 ms after the end of the saccade, ‘x’ and ‘y’ indicate respectively these two periods of fixation. Lower blue trace: horizontal conjugate position. Upper orange trace: horizontal disconjugate position.

Horizontal eye movements were defined using the velocity of the signal, respectively conjugate velocity for saccades and disconjugate velocity for vergence. The onset, or offset, were marked as the time when velocity signal exceeded, or dropped respectively below 10% of the maximum velocity. Similar criteria have been used in several other studies (Bucci *et al*.^36^, Yang and Kapoula^37^, Vernet *et al*.^38^): *i* for the onset and *p* for the offset of each eye movement (see Fig. 1). The automatic position of the markers was carefully verified by visual inspection of the individual eye movement traces. From these markers, we measured the amplitude of the movement (between *p* and *i*).

### Saccades analysis

We focused our analysis on progressive saccades, i.e. from left to right occurring in the time course of the experiment; regressive or corrective saccades in the opposite direction were scars (<5%). A few markers were added from the saccades analysis, *x* and *y* respectively 80 ms and 160 ms after *p*, as post-saccadic fixation marks (see Fig. 1). From these markers, we measured the amplitude of the saccades, the saccade disconjugacy, the amplitude of the post saccadic drifts during the first 80 ms (between *x* and *p*) and the first 160 ms (between *y* and *p*) of fixation, using the conjugate signal and the disconjugate signal.

Additionally, we evaluated fixation disparity during the beginning of each fixations and considering 3 critical points: the end of the saccade (“p”), 80 ms (“x”) and 160 ms (“y”) after the end of the saccade. These time points correspond to the two-time constant of fixation drift (see Kapoula *et al*.^39^). We measured the fixation disparity observed on these three points by subtracting the value of the vergence angle measured from the values of the vergence angle expected at 50 cm: a positive value shows a higher vergence angle than expected (esodisparity), and a negative value shows a smaller vergence angle than expected (exodisparity). Using these three values, we calculated the mean values of fixation disparity for each fixation. Because of the lack of monocular calibration, fixation disparity values would not be accurate^40^. Nevertheless, we decided to use the individual mean values of the standard deviation as an indicator to evaluate the variability of the calculated fixation disparity, which allows comparisons with earlier studies in this field^41,42^.

We were interested in evaluating how saccade motor control and related post saccadic drift influence fixation disparity in 3 different conditions. Indeed, Vernet *et al*.^38^ showed that the post saccadic disconjugate drift may act to reduce the saccade disconjugacy very early during the fixation (48 ms after the end of the saccade during reading). For all these reasons, we evaluated the fixation disparity for the same period, i.e. the beginning of the fixation, regardless of the total duration of the fixation that can be lengthened according to the task specificity.

**Figure 2:**
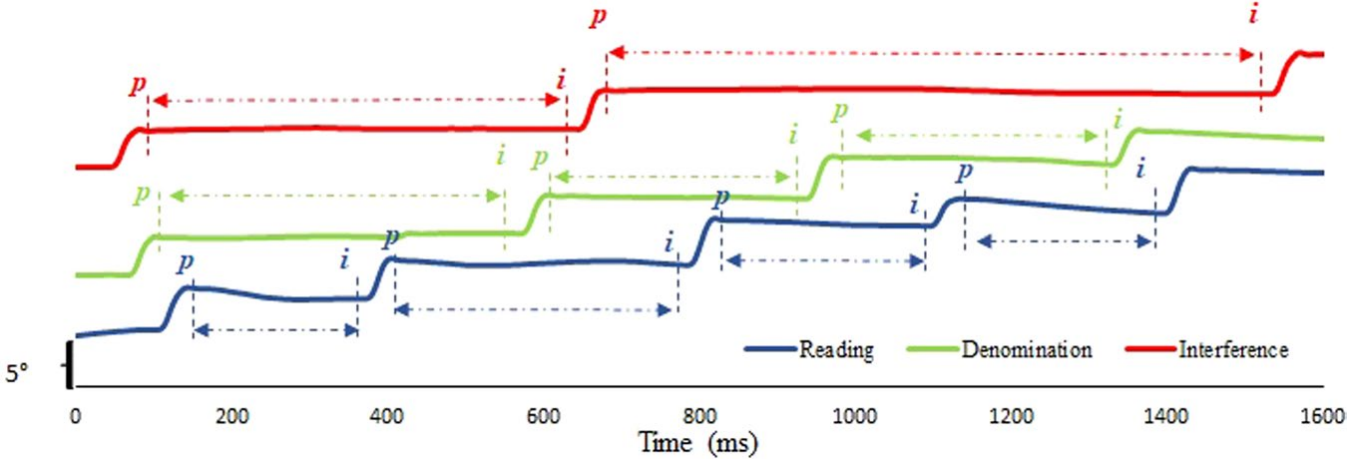
Evolution of the conjugate signal of the same subject (s. 17) when accomplishing the reading task (blue trace), the denomination task (green trace) and the interference task (red trace). Fixation duration are indicated between the end of the saccade (‘p’) and the beginning of the next one (next ‘i’).

We also calculated the fixation duration (between *p* and the *i* for the next saccade, see Fig. 2).

For 20 subjects, 90 to 95% of trials were used for statistical analysis, 5 to 10% were rejected due essentially to blinks or partial lost signal during the recording, especially concerning the fixation duration parameter; for one subject in one denomination trial and one interference trial, 30% of the saccades were rejected due to a loss of signal for one eye during the recording. It is important to note that two subjects reported prismatic distortion during the Prism condition but did not experiment double vision, and two other subjects reported headaches after the Lens condition but did not reported blurred vision.

### Stroop tests results

We used similar methods to those used by Daniel and Kapoula^10^. Time, corrected errors (when the subject made a mistake but corrected it immediately after) and uncorrected errors were measured for each task and for each subject. To evaluate the flexibility between tasks in Stroop and the impact of an induced vergence/accommodation conflict, we also calculated the time differences in each condition using the global time duration and the mean values in fixation duration. According to MacLeod^7^ and Jensen *et al*.^43^, time differences are believed to be more appropriate to evaluate Stroop interference. As in the study of Stuss *et al*.^44^, we opted for the following formulas, using the global time duration and the mean values of the fixation duration:

$${\rm{Interference}}\,{\rm{Effect}}\,({\rm{IE}})={\rm{Interference}}-{\rm{Denomination}}$$

The Error Rate (ER) gives information on the capacity of the subject to not make mistakes during the different tasks, especially the interference task. The higher it is, the more the subject made errors during the task, which gives information on the distractibility during the test. We calculated it with this formula:

$${\rm{ER}}={\rm{numbers}}\,{\rm{of}}\,{\rm{corrected}}\,{\rm{errors}}+({\rm{numbers}}\,{\rm{of}}\,{\rm{uncorrected}}\,{\rm{errors}}\times {\rm{2}})$$

A corrected error (when the subject made a mistake but corrected it immediately after) had to represent a lower importance than an uncorrected one. Such weighting is usually applied to clinical use of the test (Victoria test adapted for French), as uncorrected errors may represent higher loss of attention.

### Statistical analysis

#### Eye movement’s parameters and the Stroop test

this study provides for the first time a recording of the eye movements during the different tasks of the Stroop test. As targets were equally spaced and the eye movement demand was similar but each task (reading, denomination and interference) implicates a different level of cognitive demand, we expected to find different binocular coordination behaviors depending on the task. We first focused on the Control condition results, searching for an effect of the cognitive task (reading, denomination and interference) on the motor (amplitude, saccade disconjugacy, post saccadic drifts) and the temporal (fixation duration) parameters of the saccades and fixations, using the individual data. We used a non-parametric Friedman ANOVA as the number of subjects was limited. When a significant main effect was found, a post-hoc using non-parametric Wilcoxon test was then performed to compare tasks ‘results two by two. As the saccade disconjugacy can be corrected by the post saccadic disconjugacy drift (see Vernet *et al*.^38^), we also searched for a possible correlation between the amplitude of the saccade disconjugacy and the amplitude of the post saccadic disconjugacy drift. We used therefore a Spearman correlation analysis on the individual mean values.

#### Induced accommodation/vergence conflict

we investigated the effect of the induced convergence/accommodation conflict on the saccades and fixations parameters. As the Stroop test is made of three different tasks, we searched for an effect of a lens-induced (Lens condition) or prism-induced (Prism condition) conflict on the motor parameters (amplitude, speed, saccade disconjugacy, post saccadic drifts) and the temporal parameter (fixation duration) of the saccades in the reading task, in the denomination task and in the interference task separately, using the non-parametric Friedman ANOVA test on individual data. When a significant main effect was found, a post-hoc using non-parametric Wilcoxon test was then performed to compare trials two by two.

We calculated separately the Interference Effect (IE), using the individual data of the global time to accomplish the entire tasks and the individual mean values of the fixation duration in each task, and the Error Rate (ER), in each condition. We applied the same analysis as described above.

## Results

### Control condition in the Stroop test: cognitive demand and eye movement’s parameters

#### Amplitudes of the saccades

The non-parametric Friedman ANOVA revealed a significant effect of the task on the amplitude of the saccades (X²(21, 2) = 8.67, p = 0.013). A post-hoc using non-parametric Wilcoxon tests showed significant differences between Reading and Interference (3.69 ± 0.29° vs 3.51 ± 0.29°; Z = 2.59, p = 0.0096) and between Interference and Denomination (3.51 ± 0.29° vs 3.65 ± 0.29°; Z = 2.41, p = 0.016). No significant difference was found between Reading and Denomination (3.69 ± 0.29° vs 3.65 ± 0.29°; Z = 0.99, p = 0.32).

#### Fixation duration following the saccades

The non-parametric Friedman ANOVA revealed a significant effect of the task on the length of the fixation duration (X²(21, 2) = 42, p < 0.0001). A post-hoc using non-parametric Wilcoxon tests showed significant differences between Interference and Reading (543.45 ± 90.04 ms vs 327.59 ± 87.58 ms; Z = 4.01, p = 0.00006), between Interference and Denomination (543.45 ± 90.04 ms vs 432.01 ± 61.85 ms; Z = 4.01, p = 0.00006) and between Reading and Denomination (327.59 ± 87.58 ms vs 432.01 ± 61.85 ms; Z = 4.01, p = 0.00006).

#### Saccade disconjugacy

The non-parametric Friedman ANOVA did not reveal any significant effect of the task, neither on the algebraic value of disconjugacy (X²(21, 2) = 0.095, p = 0.95) nor on the absolute value (X²(21, 2) = 0.286, p = 0.87) of the saccades.

#### Fixation disparity

The non-parametric Friedman ANOVA did not reveal a significant effect of the task, neither on the algebraic value of the amplitude of the fixation disparity (X²(21, 2) = 4.095, p = 0.13), nor on the mean values in standard deviation (X²(21, 2) = 2.95, p = 0.23).

#### Saccade disconjugacy and the following post-saccadic drift

**Figure 3:**
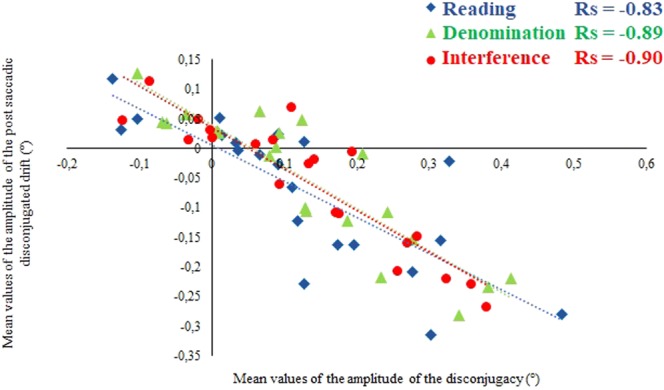
Linear regression plot of the amplitude of the following post-saccadic disconjugacy in degrees (mean values, °) measured 80 ms after the end of each progressive reading saccade as a function of the amplitude of the saccade disconjugacy in degrees (mean values, °), concerning the reading task (blue diamonds), the denomination task (green triangles) and the interference task (red dots) in the Control condition. Spearman Rs correlation coefficient are indicated.

significant correlations (p < 0.01) were found for each task and in each condition analyzing the linear regression of the amplitude of the saccade disconjugacy as a function of the amplitude of the post saccadic disconjugate drift after 80 ms when using the mean values, as shown on Fig. 3. These results indicate that the post saccadic drift may act to reduce the misalignment of the eyes at the end of the saccade (see Vernet *et al*.^38^), and the quality of this relation appears to be similar for the different tasks of the Stroop test. A higher correlation coefficient is found for Interference (r_s_ = −0.90) compared to Reading (r_s_ = −0.83) and Denomination (r_s_ = −0.89, see Fig. 3).

To determine if the correlation coefficients were significantly different, we applied a comparison of nonoverlapping correlations based on dependent groups two by two. The Silver, Hittner, and May’s modification of Dunn and Clark’s z using a back transformed average Fisher’s Z procedure did not reveal a significant difference between the Reading task and the Denomination task (z = 1.3339, p = 0.1822), between Reading and Interference (z = 1.3709, p = 0.1704), nor between Denomination and Interference (z = 0.2859, p-value = 0.7750).

To summarize, the results show that the cognitive load has a major impact on the fixation duration, but not on the binocular motor control of the disconjugacy of the saccades and the fixation disparity. However, small saccades were more frequent during the interference task, and this could explain the significant smaller amplitude of the saccades, which suggest a different strategy of exploration for this task compared to the others. The binocular coordination of saccades and the stability of the fixation appear to not have been altered by the higher cognitive demand of the interference task (see Table 1).

**Table 1:** Group mean values (bold type) and SD of the results concerning saccades and fixations parameters in the control condition during the different tasks of the Stroop test (Reading, Denomination and Interference). Significant differences depending on the task (p < 0.05) are first indicated with an asterisk.

	Reading task	Denomination task	Interference task
Amplitude of the saccades (°)	3.69* ^**bc**^	3.65* ^**bc**^	3.51* ^**bc**^
SD	±0.29	±0.29	±0.30
Fixation duration (ms)	327.59* ^**abc**^	432.01* ^**abc**^	543.55* ^**abc**^
SD	±54.88	±61.85	±90.04
Saccade disconjugacy (°) Algebraic value	**0.12**	**0.13**	**0.13**
SD	±0.16	±0.15	±0.15
Standard deviation in fixation disparity (°)	**0.38**	**0.38**	**0.43**
SD	±0.19	±0.13	±0.19

^a^Significant difference between the Reading task and the Denomination task results.

^b^Significant difference between the Denomination task and the Interference task results.

^c^Significant difference between the Reading task and the Interference task results.

### Temporal analysis and associated Stroop test performances

#### Global time duration and fixation duration

**Figure 4:**
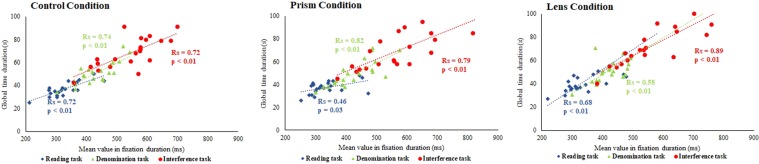
Linear regression plot of the global time to accomplish the task in seconds (s) as a function of the mean values of fixation duration in milliseconds (ms); each point is an individual value in the Control condition, the Prism condition and the Lens condition. Values concerning Reading (blue diamonds), Denomination (green triangles) and Interference (red dots) are reported. Spearman Rs correlation coefficient and p values are indicated for each task in the same color.

As reading saccades are small and fast to execute, fixations represent the major part of time in which the executive process involved by the Stroop tasks take place. We performed a Spearman correlation analysis between the mean values of fixation duration and the global time. As expected, the two measures were significantly and positively correlated for each Stroop task, for each condition and for all subjects (see Fig. 4).

Thus, the mean value of fixation duration reflects the process involved by each task of the Stroop test. Note that we measured fixation durations following the progressive saccades, without taking fixations following the regressive saccades due to errors or hesitation, to obtain a more precise measure of cognitive process. We therefore decided to use the fixation duration mean values to also calculate the usual Stroop interference effect (see Tables 2 and 3).

**Table 2:** Mean values concerning the global time to accomplish the different Stroop tasks in seconds, depending on the condition.

Task	Condition	Mean value in global time (s)	Friedman’s ANOVA results	Post-hoc Wilcoxon tests results
Reading	Control	36.90 ± 6.13	**X²(21, 2) = 12.34** p = 0.002*	−C vs P: Z = 2.98, p = 0.003* −P vs L: Z = 2.48, p = 0.013*
	Prism	42.95 ± 17.89		
	Lens	37.24 ± 5.59		
Denomination	Control	50.95 ± 9.81	X²(21, 2) = 0.48 p = 0.79	
	Prism	54.10 ± 17.24		
	Lens	49.67 ± 11.46		
Interference	Control	68.19 ± 13.44	X²(21, 2) = 3.58 p = 0.17	
	Prism	76.67 ± 24.31		
	Lens	70.05 ± 16.89		
Stroop Interference Effect	Control	17.24 ± 9.24	**X²(21, 2) = 6.10** p = 0.047*	−C vs P: Z = 2.26, p = 0.023*
	Prism	22.57 ± 11.48		
	Lens	20.38 ± 11.80		

Associated calculation of the Stroop interference effect is indicated, as Friedman’s ANOVA and post-hoc Wilcoxon tests results. Significant differences are written in bold type.

**Table 3:** Mean values concerning fixation duration in the different Stroop tasks (ms), depending on the condition.

Task	Condition	Mean value in fixation duration (ms)	Friedman’s ANOVA results	Post-hoc Wilcoxon test results
Reading	Control	327.6 ± 54.9	X²(21, 2) = 2.00 p = 0.37	
	Prism	345.2 ± 83.2		
	Lens	335.7 ± 59.8		
Denomination	Control	432.0 ± 61.9	X²(21, 2) = 0.38 p = 0.83	
	Prism	440.4 ± 96.3		
	Lens	425.3 ± 71.8		
Interference	Control	543.6 ± 90.0	X²(21, 2) = 4.57 p = 0.101	
	Prism	594.5 ± 158.9		
	Lens	574.2 ± 122.3		
Stroop Interference Effect	Control	111.5 ± 67.5	**X²(21, 2) = 12.09** p = 0.0023*	−C vs P: Z = 2.87, p = 0.004* −C vs L: Z = 2.21, p = 0.027*
	Prism	154.1 ± 112.2		
	Lens	148.9 ± 76.4		

Associated calculation of the Stroop interference effect is indicated, as Friedman’s ANOVA and post-hoc Wilcoxon tests results. Significant differences are written in bold type.

Inspection of the Tables 2 and 3 shows that for both total time and fixation duration, statistical significant effect of the conditions occurs concerning the interference effect, particularly when fixation duration is used: the interference effect is much higher in the prism and the lens condition relative to the control condition.

#### Error rate

Concerning the Interference task, the non-parametric Friedman’s ANOVA revealed a significant effect of the conditions on the ER (X²(21, 2) = 7.65, p < 0.022). A post-hoc using non-parametric Wilcoxon tests showed a significant difference between the Control condition and the Prism condition (1.71 ± 1.42 vs 3.57 ± 3.20; Z = 2.11, p = 0.035). Concerning the difference between the Lens condition and the Prism condition, the results tended to be similar (1.76 ± 1.79 vs 3.57 ± 3.20; Z = 1.89, p = 0.059) but did not reach a significant level.

To summarize, results show that the Stroop performances were stable in the Control condition compared to the induced vergence and accommodation conflict conditions. However, when calculating the interference effect, which reflects the time consuming in inhibiting the reading answer during the interference task, subjects showed more difficulties to accomplish the test in the induced conflict conditions, especially in the Prism condition. Prism induced conflict appears to have a higher impact on the Stroop performance, as the error rate was also higher in this condition.

### Impact of an induced accommodation/vergence conflict on saccades and fixations parameters

Mean values and standard deviations of the saccade and fixations parameters measured during the different tasks and conditions are shown on Tables 4–6.

**Table 4:** Mean values and standard deviation concerning saccades and fixation parameters during the Reading task of the Stroop test. Friedman’s ANOVA and post-hoc Wilcoxon tests results are indicated.

Parameter	Condition	Mean value in fixation duration (ms)	Friedman’s ANOVA results	Post-hoc Wilcoxon test results
Amplitude of the saccades (°)	Control	3.69 ± 0.29	X²(21, 2) = 1.14 p = 0.56	
	Prism	3.65 ± 0.33		
	Lens	3.70 ± 0.33		
Saccade disconjugacy (°) *algebraic value*	Control	0.12 ± 0.16	**X²(21, 2) = 10.57** p = 0.005*	−P vs L: Z = 3.42, p = 0.0006* −P vs C: Z = 1.96, p = 0.049*
	Prism	0.06 ± 0.17		
	Lens	0.16 ± 0.18		
Saccade disconjugacy (°) *absolute value*	Control	0.20 ± 0.13	X²(21, 2) = 0.67 p = 0.72	
	Prism	0.21 ± 0.10		
	Lens	0.23 ± 0.13		
Post saccadic disconjugate drift (°) After 80 ms	Control	−0.07 ± 0.12	**X²(21, 2) = 9.52** p = 0.0085*	−P vs C: Z = 2.24, p = 0.025* −P vs L: Z = 3.42, p = 0.0006* −L vs C: Z = 2.38, p = 0.017*
	Prism	−0.03 ± 0.13		
	Lens	−0.08 ± 0.12		
Post saccadic disconjugate drift (°) After 160 ms	Control	−0.10 ± 0.15	**X²(21, 2) = 9.52** p = 0.0085*	−P vs C: Z = 2.17, p = 0.029* −P vs L: Z = 2.79, p = 0.005*
	Prism	−0.05 ± 0.14		
	Lens	−0.11 ± 0.15		
Standard deviation in fixation disparity (°)	Control	0.38 ± 0.19	**X²(21, 2) = 11.14286** p = 0.0038*	−P vs C: Z = 3.0065, p = 0.0026* −P vs L: Z = 2.172, p = 0.00298*
	Prism	0.56 ± 0.26		
	Lens	0.45 ± 0.27		

Significant differences are written in bold type.

**Table 5:** Mean values and standard deviation concerning saccades and fixation parameters during the Denomination task of the Stroop test.

Parameter	Condition	Mean value in fixation duration (ms)	Friedman’s ANOVA results	Post-hoc Wilcoxon test results
Amplitude of the saccades (°)	Control	3.65 ± 0.29	X²(21, 2) = 2.95 p = 0.23	
	Prism	3.74 ± 0.35		
	Lens	3.74 ± 0.37		
Saccade disconjugacy (°) *algebraic value*	Control	0.13 ± 0.15	**X²(21, 2) = 7.24** p = 0.026*	−P vs C: Z = 2.42, p = 0.016* −P vs L: Z = 2.28, p = 0.023*
	Prism	0.07 ± 0.22		
	Lens	0.13 ± 0.18		
Saccade disconjugacy (°) *absolute value*	Control	0.20 ± 0.11	X²(21, 2) = 3.52 p = 0.17	
	Prism	0.22 ± 0.13		
	Lens	0.23 ± 0.11		
Post saccadic disconjugate drift (°) After 80 ms	Control	−0.05 ± 0.12	**X²(21, 2) = 12.67** p = 0.0018*	−P vs C: Z = 2.41, p = 0.016* −P vs L: Z = 3.006, p = 0.003*
	Prism	−0.02 ± 0.12		
	Lens	−0.07 ± 0.13		
Post saccadic disconjugate drift (°) After 160 ms	Control	−0.08 ± 0.14	X²(21, 2) = 4.95 p = 0.08	
	Prism	−0.04 ± 0.14		
	Lens	−0.08 ± 0.15		
Standard deviation in fixation disparity (°)	Control	0.38 ± 0.13	**X²(21, 2) = 11.809** p = 0.00273*	−P vs C: Z = 3.46, p = 0.0005* −P vs L: Z = 2.42, p = 0.016*
	Prism	0.63 ± 0.35		
	Lens	0.44 ± 0.24		

Friedman’s ANOVA and post-hoc Wilcoxon tests results are indicated. Significant differences are written in bold type.

**Table 6:** Mean values and standard deviation concerning saccades and fixation parameters during the Interference task of the Stroop test. Friedman’s ANOVA and post-hoc Wilcoxon tests results are indicated.

Parameter	Condition	Mean value in fixation duration (ms)	Friedman’s ANOVA results	Post-hoc Wilcoxon test results
Amplitude of the saccades (°)	Control	3.51 ± 0.30	X²(21, 2) = 0.67 p = 0.72	
	Prism	3.48 ± 0.33		
	Lens	3.55 ± 0.36		
Saccade disconjugacy (°) *algebraic value*	Control	0.13 ± 0.15	X²(21, 2) = 3.43 p = 0.18	
	Prism	0.06 ± 0.20		
	Lens	0.11 ± 0.17		
Saccade disconjugacy (°) *absolute value*	Control	0.20 ± 0.11	X²(21, 2) = 0.67 p = 0.72	
	Prism	0.22 ± 0.11		
	Lens	0.21 ± 0.10		
Post saccadic disconjugate drift (°) After 80 ms	Control	−0.06 ± 0.11	**X²(21, 2) = 10.29** p = 0.0058*	−P vs C: Z = 2.69, p = 0.007* −P vs L: Z = 3.18, p = 0.001*
	Prism	−0.03 ± 0.12		
	Lens	−0.06 ± 0.12		
Post saccadic disconjugate drift (°) After 160 ms	Control	−0.08 ± 0.14	**X²(21, 2) = 12.09** p = 0.002*	−P vs C: Z = 3.15, p = 0.0017* −P vs L: Z = 3.08, p = 0.002*
	Prism	−0.04 ± 0.13		
	Lens	−0.09 ± 0.14		
Standard deviation in fixation disparity (°)	Control	0.43 ± 0.19	**X²(21, 2) = 6.381** **p = 0.041**	−P vs C: Z = 2.73, p = 0.006*
	Prism	0.59 ± 0.26		
	Lens	0.51 ± 0.29		

Significant differences are written in bold type.

Inspection of the Tables 4–6 shows that prism induced vergence-accommodation conflict appears to have a higher impact on binocular motor control of the saccades in most of the Stroop tasks, as the saccade disconjugacy and the disconjugate drift values modulate in the Prism condition compared to the Control and the Lens conditions. The mean values in standard deviation concerning fixation disparity, reflecting the stability of the fixation, were also statistically higher in the Prism condition, which could also demonstrate the deterioration of the correlation between the amplitude of the saccade disconjugacy and the post saccadic disconjugate drift associated, especially when the cognitive demand is high (interference task). The Lens induced conflict shown minor impacts on disconjugate drift, however the results differ as a function of the task studied, suggesting a lower impact on binocular motor control of the saccades and fixation.

#### Saccade disconjugacy and the following post saccadic disconjugacy drift

**Figure 5:**
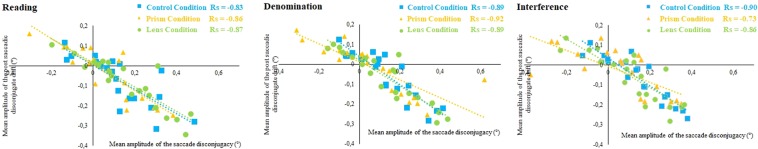
Linear regression plot of the amplitude of the post-saccadic disconjugacy drift in degrees (°) measured 80 ms after the end of each progressive reading saccade as a function of the amplitude of the intra-saccadic disconjugacy in degrees (°) concerning Reading, Denomination and Interference. Mean values concerning the Control condition (blue squares), the Minus lenses condition (green dots) and the Prisms condition (orange triangles) are reported for each task. Spearman Rs correlation coefficient are indicated in bold type.

significant correlations (p < 0.01) were found for each task and in each condition analyzing the linear regression of the amplitude of the saccade disconjugacy as a function of the amplitude of the post saccadic disconjugate drift after 80 ms using the individual mean values, as shown on Fig. 5.

To determine if the correlation coefficients were significantly different, we applied a comparison of nonoverlapping correlations based on dependent groups two by two. The Silver, Hittner, and May’s modification of Dunn and Clark’s z using a back transformed average Fisher’s Z procedure results are shown on Table 7.

**Table 7:** Statistical comparisons of the correlations coefficient two by two for each task.

Task	Condition	Correlation coefficients	z and p-value
Reading	Control vs Prism	−0.83 vs −0.86	z = 0.4187, p-value = 0.6754
	Control vs Lens	−0.83 vs −0.87	z = 0.4837, p-value = 0.6286
	Prism vs Lens	−0.86 vs −0.87	z = 0.0413, p-value = 0.9670
Denomination	Control vs Prism	−0.89 vs −0.92	z = 0.5688, p-value = 0.5695
	Control vs Lens	−0.89 vs −0.89	z = 0.0580, p-value = 0.9538
	Prism vs Lens	−0.92 vs −0.89	z = −0.4799, p-value = 0.6313
Interference	Control vs Prism	−0.90 vs −0.73	z = −2.0878, p-value = 0.0368*
	Control vs Lens	−0.90 vs −0.86	z = −0.7634, p-value = 0.4452
	Prism vs Lens	−0.73 vs −0.86	z = 1.4716, p-value = 0.1411

## Discussion

### The Stroop test and properties of eye movements

To our knowledge, the properties of eye movements during the different Stroop tasks has never been studied before. The Stroop test is a golden neurological test used in neuroscience, in developmental studies including dyslexia^45^, in aging and neurodegenerative diseases to evaluate cognitive executive functions^46,47^. Indeed, the Stroop test is believed to stimulate attentional and inhibitory mechanisms, particularly the interference task in which reading must be inhibited to name the color of the written words. This task also bears the greatest cognitive load when compared to the reading and the denomination ones. The first question we asked was: what is the effect of interference or color effect on eye movement properties? It is known from clinical studies that the response time is increased during the color denomination and the interference tasks relative to simple reading task but this increase of time could be related to several potential factors. As such, this study sought to establish which component exactly is related to this increase of time. We have shown that the length of timing while naming the color dots or doing the interference task is mainly due to increase of fixation duration. Fixation duration is the time during which the central nervous system processes visual information to name the color of the ink the word is written. It is possible that this cognitive task of interference is not entirely executed during the period of fixation, and partially this process can be continued and achieved at the beginning of the next saccade; still the results clearly indicate that fixation duration is the time during which this interference process occurs primarily. So, the interference task involves longer fixation durations and this result is in line with literature from other fields: e.g. fixation durations during reading are believed to related to cognitive processing^48–50^.

At the motor site, it is important to note the increase of the frequency of the small saccades (<1.5°) which leads to the overall decrease of the mean amplitudes of the saccades during the interference test, and the increase of regressive saccades. This behavior is to our knowledge reported for the first time. It is evocative of the presence of a strategy consisting in scrutinizing carefully every item during the interference task. It is possible that micro saccades, that represent focus scanning^51^, are more frequent in the interference task but this needs further investigation. However, the results showed that the properties of the saccades themselves remained stable whatever the cognitive task: the coordination of the saccades, the amplitude and variability of the fixation disparity along with the correlation coefficients between the amplitude of the saccade disconjugacy and the post saccadic drift associated, remained similar in the different tasks of the Stroop test (see Table 1 and Fig. 4).

In conclusion, we demonstrate here for the first time strong modulation of fixation duration by the cognitive demand of the Stroop test: the measure of fixation duration reflects in an incremental way the degree of difficulty of the test. Naming the color during the denomination task requires longer fixation because is less automatic than reading. The interference task requires inhibition of the reading response plus the color naming response, which involves higher cognitive processes.

### Interplay between cognition and vergence/accommodation conflict

Inducing an accommodation/vergence conflict by adding prisms or adding spherical lenses involves shifting of the accommodation or the convergence demand, to maintain single and clear vision. The conflict creates a stress on the visual system, as the accommodative response does not correspond anymore to the vergence response. Even if the visual system can partially compensate this mismatch (as shown in adults^52^), this effort in compensation itself for restoring could require visual attention resources and impact on cognitive processes. It is important to note that our selected subjects had to wear their usual correction. However, even if the refraction was checked previously, objectively and subjectively using the monocular fogging method to a standard endpoint of maximum plus, small amount of residual hyperopia could remain as refraction was not determined under cycloplegic conditions. Therefore, it is important to acknowledge that the Lens condition could have produced more difficulties for some of the subjects.

In the present study, induction of an accommodation or vergence conflict with prism or with spherical lenses significantly increases the interference effect during the Stroop tasks, and the increase is more significant for the prism-induced conflict than for the lens-induced conflict. This result sheds new light on the interplay between vergence or accommodation conflict, cognition and eye movement control. The interference effect measured by the difference in fixation duration between the interference and color denomination tasks is on average 42 milliseconds with the prisms on and 37 milliseconds with the spherical lenses on; this is indicative of the importance of high quality visual input when high cognitive executive function are in process. Note that the neural circuits that process vergence disparity and accommodation signals (visual cortex, parietal and frontal lobes)^21–26^ are partially the same as they are ones that control cognition (e.g. frontal and parietal lobes)^14,15,53–58^. Indeed, this increase could be explained by the fact that attentional resources to treat the disparity induced by the prisms or the blur induced by the lenses are required and thereby diminishing the availability of such resources for the cognitive task (i.e. inhibition of reading and naming of the color). In other words, tolerance to the vergence/accommodation conflict seems to be lower in the case of the interference task, reflected by the substantial fixation duration increase. The visual stress induced by the conflict conditions forced the subjects to immediately distribute their cognitive resources to maintain single and clear vision. This redistribution involves sharing of cognitive attention resources required by the Stroop test, thereby decreasing their performances.

Another important point of our results is that the prism-induced conflict appears to be more disturbing than the lens-induced conflict. Minus lenses will induce blur and force the subject to accommodate for recovering clear vision, shifting positively the accommodative response while the convergence demand stays the same; the prisms will induce a disparity error, will cause convergence increase and will conflict with the accommodation demand. The conflict between accommodation and vergence can be higher in the Prisms condition on motor parameters, than in the Lens condition. As shown by Bharadwaj *et al*.^52^, the visual system usually shows a larger tolerance concerning the accommodative response to a Lenses condition than concerning the vergence response to a Prisms condition. This also could be attributable to the fact that the size of the Panum’s area are smaller than the depth-of-focus, which allows a larger tolerance in the accommodative response during the lens-induced condition. Thus, avoiding double vision through prisms requires the visual motor system to produce an adequate, precise and stable vergence response, compared to the lens-induced blur on the accommodative response. Also, it is possible that neural circuits dealing with blur and accommodative cues might be less interfering with cognition than circuits controlling disparity eye movements. Vergence and accommodative adaptation involved the cerebellum for such responses^22,59^. In other words, disparity of images is more interfering with cognition than blurred images, and this is quite plausible physiologically as disparity calls for immediate adjustment of vergence to avoid diplopia while blurred images can be tolerated^60^. The higher interference with cognition was evident both, in terms of increase of fixation duration but also in terms of significantly higher error rates in the interference task in the prism condition compared to the lenses or the normal condition. The higher error rate reinforces the conclusion that the Prisms condition has a higher impact on cognitive resources. Thus, we hypothesize that this involves redistribution of these resources in the extended neural network (visual-frontal-parietal-cerebellum), that subtends cognitive and visual aspects.

Nevertheless, the Stroop interference is linked to two different parameters: the speed of word reading and the efficiency of the inhibitory mechanism that must block the reading response^9^. As reading was not lengthened in the Lenses condition but interference effect was higher than in the control condition, we argue that the vergence/accommodation conflict impacted mainly the inhibitory mechanism. As the reading task was slightly lengthened in the Prisms condition compared to the other conditions, it also indicates that reading processes could have been destabilized, which could be responsible of a higher increase of the interference effect with the prisms on. Yet, the higher error rate in the interference task shows a more likely lower efficiency of inhibition. Based on such observations, we argue that accommodation/vergence conflict interferes mainly with the inhibition process involved in the Stroop test.

### Accommodation/vergence conflict modulates saccade disconjugacy and fixation disparity

In line with our general theoretical concept according to which binocular coordination of saccades depends on the quality of vergence and its synergy with accommodation^10^, we expected the induced conflict to modify the disconjugacy of the saccades. The parameters that first did change are the quality of binocular coordination of the saccades and the quality of the link between binocular coordination of the saccade and binocular coordination of the drift of the eyes during the fixation period. The absolute value of saccade disconjugacy did not change significantly; yet, the algebraic value of the intra-saccadic disconjugacy became more negative, which means that in the presence of the prisms only and in almost all three tasks, saccade disconjugacy was more frequently divergent. Thus, the prisms act on the intra-saccadic disconjugacy. Another important aspect is the weakening of the correlation between intra-saccadic disconjugacy and the disconjugacy during the following fixation. Previous studies showed significant correlation between these two parameters^38,61^; such correlation was associated with a better capacity for reducing the disparity during fixation that results from the intra-saccadic disconjugacy. In the present study, the prisms altered this correlation that became weaker than in the lenses and in the control condition, and this for the interference Stroop task (see Fig. 5). Thus, prisms caused a deregulation of the capacity of the central nervous system to control the sequence of intra-saccadic and post-saccadic disconjugacy, as the standard deviation of the fixation disparity appeared also to be highly impacted in the prism condition compared to the others, and this for most of the Stroop tasks. We argue that this is destructive and could also interfere with cognitive executive functions.

However, if the coordination of the saccade was not disrupted in the Lenses condition, the amplitude and the variability of the fixation disparity values showed a destabilization: maintaining the appropriate vergence angle during the fixation was more difficult in the Prisms and in the Lenses conditions. And, this critical phase of fixation is essential for single vision, as it permits reading and cognitive processes to occur. A higher disparity at the beginning of the fixation must therefore be reduced to maintain the vergence angle stable. In addition to a poorer saccade coordination, we make the hypothesis that a higher and a more variable fixation disparity can interfere with the efficiency of the inhibition processes as tested in the Stroop test.

## Conclusion

In conclusion, this study demonstrates that vergence or accommodation conflicts, particularly such as induced by prisms, interferes with cognitive executive functions stimulated by the Stroop test. Cognitive interference is reflected mainly by longer fixations and higher rates of errors. The mismatch induced by prisms also alters the disconjugacy of saccades that becomes more divergent, and the correlation between intra-saccadic disconjugacy and the post-saccadic disconjugacy drift weakens. Therefore, residual disparities during fixation occur and this would interfere with cognition. The study also suggests better tolerance to the mismatch due to blur induced by spherical lenses. This study has both theoretical and clinical implications: (1) at the theoretical level, the interplay between vergence/accommodation conflict and cognition is of interest, as visuo-motor and cognition processes rely on same parietal/frontal cortical structures; (2) at the clinical level, it is important to consider that tolerance to vergence/accommodation mismatch depends upon the difficulty of the cognitive tasks: tolerance is lower when higher executive functions such as those of the interference task are in progress.
